# New Drugs, Appliances, and Things Medical

**Published:** 1890-07-19

**Authors:** 


### NEW DRUGS, APPLIANCES, AND THINGS MEDICAL.

[All preparations, appliances, novelties, etc., of which a notice is desired, should be sent to The Editor, The Lodge, Porchester Square, W.]

### THE EXCELSIOR BED-LIFT (MONKHOUSE'S PATENT).

We have examined the above, and consider it a most valuable invention. It is intended for lifting and turning heavy or helpless patients, and for use in all cases in which the draw-sheet is necessary—*i.e.*, whenever the utmost care is required in moving invalids. The nursing in such cases as paralysis, rheumatic fever, bad accidents, and fractures, and also in the lying-in room will be greatly facilitated by its use. Among its advantages may be enumerated—(1) *Its Safety*—We tested this thoroughly, and have convinced ourselves that it is perfectly strong and safe. (2) *Its Position*—It does not overhang the patient, so does not alarm by its strange or oppressive appearance. (3) *The Ease with which it can be Worked*—One person can lift the heaviest patient with ease and comfort to both operator and sufferer. (4) *Its Adaptability*—It can be fitted to any iron bedstead, and when fitted is always ready for use. The winding apparatus is under the bed, and out of the way of the lift. (5) By its use beds can be made and aired, the back of the patient sponged, or dressed if there are bed sores, and it greatly tends by its use to prevent the formation of these last. Also a patient can be turned or raised, and firmly supported at any angle. By detaching the lifting ropes, the patient can be lifted off the bed, and carried as in a stretcher. Thus one lifter with a series of webs will do for a whole ward in a hospital. The straps are placed under the patient by a very ingenious arrangement, after the fashion of the pocket on a Barnes' bag. They are fixed also to the frame by a very simple but perfectly safe cross-pin. In cases where a bath is necessary, by placing this on the bed (a special indiarubber bath can be supplied), the patient can be gently lowered into it. For this purpose waterproof straps are supplied. We were greatly pleased with the apparatus. Every point has been thought out and perfected, and the whole reflects great credit on its inventor, Mrs. Monkhouse. We notice that many of the London and provincial hospitals use it. We think it would be of great service if kept at an ambulance station in country places, or if country practitioners had one to lend out. Its cost is rather high, but its durability is great, and the ease and comfort resulting from its use will amply repay the initial expenditure.

### CAFFYN'S LIQUOR CARNIS.

In all ordinary cases of illness by far the most important item of treatment is the food. If all medical men, nurses, patients, and patients' friends would constantly recognise this fact and act upon it, the art of medicine would be able to chronicle many more and much better cures than it records at present. Food can kill, and food can cure. In the case of typhoid fever food of itself not seldom kills; in the case of certain wasting diseases raw meat juice or some similar food is all that is required for a cure. Of meat juices, beef teas, extracts of meat, and the like the British market is full, even to a surfeit. Why then does any man or company start a new beef juice? And why do responsible medical papers, help to bring it into notice? The Liquor Carnis of Dr. Caffyn has been unstintedly praised by most of the medical newspapers both British and Colonial. Is there any special reason for this? There is, and it is this: Liquor Carnis is not an improved meat extract, but a beef juice pure and simple. That is to say it is a substance different in kind from all the meat extracts and essences. It is not a cooked product but is perfectly raw; and it is the pure juice of beef exactly as the fluid which is squeezed out of a grape or an orange is the juice of the orange or grape. There is, of course, the addition of a small quantity of a preservative substance, without which the juice would not keep, and would therefore be practically useless.

Medical readers fully understand the value of raw meat juice. No argument is needed to convince them of that. What they want full assurance of is that any particular meat juice which they may think of ordering is the real thing itself without foreign admixture. The writer of the present article knows Dr. Caffyn and the whole history and mystery of his meat juice discovery from beginning to end. There is no doubt whatever that Caffyn's Liquor Carnis is the real Simon Pure. But no medical man need take the article on trust. All that he requires to do is to buy a bottle of the Liquor Carnis, half fill a test tube with it, and hold it over his burning spirit lamp. In a very few moments he will find in his test tube a column of solid albumen of exactly the same height as the Liquor Carnis taken. No more proof than this is wanted of the actual character and quality of the juice.

But is the Liquor Carnis digestible? And will it keep? These are the two practical questions asked by the business-like physician. It is highly digestible, and it keeps admirably; we have tried it in both particulars. It has been extensively experimented with in Australia where it was first made, and also in this country at the bedside. This is the practical test of its medicinal value. In all kinds of wasting diseases, both of children and grown up persons, its success has been very great indeed. It could not be otherwise; for we know of no food or drug possessing the same concentrated nutritive value as the juice of raw meat. It has often brought the apparently dying back to life again. Wherever raw meat juice is indicated Caffyn's Liquor Carnis should be tried. So far as we know, it is the only really raw meat juice in the market. Doctors should bear this in mind; and the public also, still more than doctors. The use of the right weapon in the fight, and of the right food or drug in the sick room, often makes all the difference between cure and death.

### SALVINE DENTIFRICE.

A sample of the above has been forwarded to us for notice. The idea is an ingenious one, and the compound pleasant to use. The dentifrice is semi-fluid, of agreeable odour and taste, and professes to be both an antiseptic and an antacid. It is put up in collapsible tubes, a great improvement, we think, in many ways on the ordinary tooth powder boxes, notably in preventing the contamination of the remaining dentifrice by the surreptitious dipping of other tooth brushes into the owner's box. Also it forms a very portable method of carrying about the highly necessary toilet accompaniment of a dentifrice, obviating the vexatious spilling of chalky powders over "best Sunday-go-to-meeting-clothes." From our examination of the contents of the tube, we consider them to be likely to fill a want, and useful in the ways claimed for them.

Erratum.

### THE DUPLEX INSTANTER THERMOMETER.

We are requested to state that this thermometer was wrongly described as the "Rapid" in our issue of the 5th inst., page 205. Mr. Hicks (8, 9, and 10, Hatton Garden) writes: "The thermometer is named and advertised as the 'Duplex Instanter.' It is also known to all the leading members of the Medical Profession by this name, and I fear, therefore, that unless you make the correction this week I shall be put to much inconvenience." We described the thermometer as the "Rapid" because it is the only thermometer known to us which enables the physician to take the temperature of the body in the least possible time. The "Duplex Instanter" thermometer mus prove a boon to busy practitioners, students, and nurses.

